# Nomograms predicting the overall survival and cancer-specific survival of patients with stage IIIC1 cervical cancer

**DOI:** 10.1186/s12885-021-08209-5

**Published:** 2021-04-23

**Authors:** Yifan Feng, Ye Wang, Yangqin Xie, Shuwei Wu, Yuyang Li, Min Li

**Affiliations:** 1 Department of Obstetrics and Gynecology, the First Affiliated Hospital of Anhui Medical University, Hefei, 230022 China; 2 Department of General Surgery, the First Affiliated Hospital of Anhui Medical University, Hefei, 230022 China

**Keywords:** Cervical cancer, Overall survival, Cancer-special survival, Nomogram, FIGO

## Abstract

**Background:**

To explore the factors that affect the prognosis of overall survival (OS) and cancer-specific survival (CSS) of patients with stage IIIC1 cervical cancer and establish nomogram models to predict this prognosis.

**Methods:**

Data from patients in the Surveil-lance, Epidemiology, and End Results (SEER) programme meeting the inclusion criteria were classified into a training group, and validation data were obtained from the First Affiliated Hospital of Anhui Medical University from 2010 to 2019. The incidence, Kaplan-Meier curves, OS and CSS of patients with stage IIIC1 cervical cancer in the training group were evaluated. Nomograms were established according to the results of univariate and multivariate Cox regression models. Harrell’s C-index, calibration plots, receiver operating characteristic (ROC) curves and decision-curve analysis (DCA) were calculated to validate the prediction models.

**Results:**

The incidence of pelvic lymph node metastasis, a high-risk factor for the prognosis of cervical cancer, decreased slightly over time. Eight independent prognostic variables were identified for OS, including age, race, marriage status, histology, extension range, tumour size, radiotherapy and surgery, but only seven were identified for CSS, with marriage status excluded. Nomograms of OS and CSS were established based on the results. The C-indexes for the nomograms of OS and CSS were 0.687 and 0.692, respectively, using random sampling of SEER data sets and 0.701 and 0.735, respectively, using random sampling of external data sets. The AUCs for the nomogram of OS were 0.708 and 0.705 for the SEER data sets and 0.750 and 0.750 for the external data sets, respectively. In addition, AUCs of 0.707 and 0.709 were obtained for the nomogram of CSS when validated using SEER data sets, and 0.788 and 0.785 when validated using external data sets. Calibration plots for the nomograms were almost identical to the actual observations. The DCA also indicated the value of the two models.

**Conclusions:**

Eight independent prognostic variables were identified for OS. The same factors predicted CSS, with the exception of the marriage status. Both OS and CSS nomograms had good predictive and clinical application value after validation. Notably, tumour size had the largest contribution to the OS and CSS nomograms.

## Background

Cervical cancer is the most common malignant tumour of the female reproductive system, and the fourth most common malignant tumour in women, after breast cancer, colorectal cancer and lung cancer [1]. In 2018, approximately 570,000 women were diagnosed with cervical cancer and 311,000 women died from it [1]. Persistent carcinogenic human papillomavirus infection is the main cause of cervical cancer development [2]. Fortunately, due to the development of the HPV vaccine, a treatment protecting against cervical precancerous lesions, the incidence and mortality of cervical cancer in developed countries are gradually decreasing [3]. However, in developing countries, cervical cancer is still one of the most common cancers and the main cause of cancer-related death in women [4]. For example, in China, the incidence and mortality of cervical cancer are increasing significantly, especially among young women [5]. Due to the substantial economic burden of cervical cancer screening and vaccination programmes, many women are still suffering from HPV infection and its related cervical cancer [6].

The International Federation of Obstetrics and Gynecology (FIGO) stage is a systematic staging system based on a clinical examination [7]. In 2018, FIGO made important adjustments to the cervical cancer staging system [7]. Compared to the 2014 FIGO staging system, several changes were introduced. (a) Horizontal infiltration width no longer affects the stage. (b) An additional stage, IB3 was added to stage IB. In the revised system for stage IB disease, for every 2 cm increase in tumour size, the substage increases. Tumours smaller than 2 cm are classified as IB1, tumours greater than or equal to 2 cm and less than 4 cm are classified as IB2, tumours greater than or equal to 4 cm are classified as IB3. (c) Pelvic lymph node metastasis or paraaortic lymph node metastasis are directly classified as stage IIIC1/2 [7, 8].

The FIGO staging system is most frequently used to assess the prognosis of patients with cervical cancer. The new FIGO stage reflects the important effect of lymph node metastasis on the prognosis of cervical cancer patients [9]. However, the survival rate is heterogeneous for patients with the same stage. The prediction of prognosis using the FIGO staging system is not sufficiently comprehensive, and the accuracy must be improved [10, 11].

A trend is to use nomograms to build cancer prediction models, because nomograms simplify a large number of complex factors into a single simple numerical estimation model to predict the probability of events [11]. Currently, few prognostic analyses of stage IIIC1 cervical cancer have been performed, and no nomogram has been established for patients with stage IIIC1 cervical cancer.

The purpose of this study was to explore the factors that affect the prognosis of patients with stage IIIC1 cervical cancer and establish nomogram models to predict the prognosis of stage IIIC1 cervical cancer.

## Materials and methods

### Data source

This retrospective observational study was conducted with data from the Surveil-lance, Epidemiology, and End Results (SEER) programme. The SEER database is a publicly available, federally funded cancer reporting system [12]. No cases extracted from the SEER database contain any personally identifying information.

One external validation set was generated to validate the nomogram in the present analysis, and the data were obtained from the First Affiliated Hospital of Anhui Medical University from 2010 to 2019; all patients in this dataset were pathologically diagnosed with cervical cancer. All patients under closed follow-up, every 3 months for the first 2 years, every 6 months for the next 3 years, and annually thereafter. This study was approved by Anhui Medical University Ethics Committee.

### Inclusion criteria

**Fig. 1:**
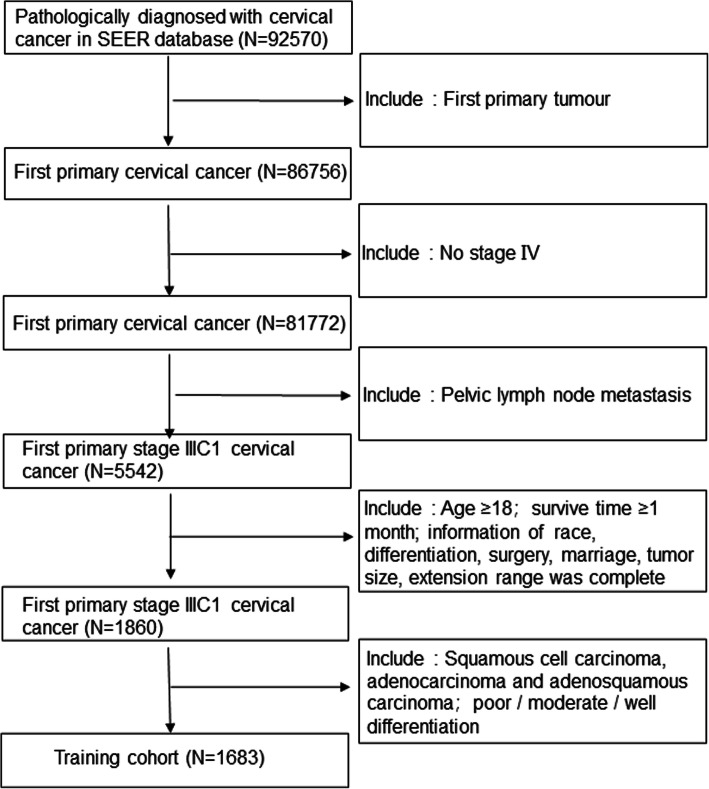
Study selection process

Inclusion criteria were patients with a pathological cervical cancer diagnosis who were included in the SEER database from 2004 to 2015; cervical cancer was the first primary tumour; no stage IV disease; all patients underwent surgery and were evaluated positive for pelvic lymph node metastasis; age ≥ 18 years; patients who died within 1 month; information about race, differentiation, surgery, marriage status, tumour size, extension range was complete; and the histopathological diagnosis was squamous cell carcinoma, adenocarcinoma or adenosquamous carcinoma utilizing ICD-O-3 codes, with poor / moderate / well differentiation (shown in Fig. 1). Patients with negative lymph nodes were selected in the same manner to compare the data with patients with pelvic lymph node metastasis. The external validation set was selected using the same criteria.

### Statistical analysis

Categorical variables are described as counts and percentages. Kaplan-Meier curves and log-rank tests were used to draw overall survival (OS) curves and cancer-specific survival (CSS) curves in groups with different lymph nodes metastasis statuses. Additionally, univariate and multivariate Cox regression models were employed to estimate hazard ratios (HRs) and exact 95% confidence intervals (CIs) to analyse the prognostic factors for stage IIIC1 cervical cancer. OS was the primary endpoint outcome from the date of diagnosis to the date of death or the latest follow-up. CSS was the special endpoint outcome from the date of diagnosis to the date of death from cervical cancer or the latest follow-up.

Significant prognostic factors of OS and CSS in the Cox proportional hazards regression model were used to build the nomograms to predict the 3- and 5-year OS and CSS rates. Harrell’s C-index and receiver operating characteristic (ROC) curves were calculated to measure the accuracy of the prediction models. Calibration plots show the relationship between the predicted probability and the actual outcome. Finally, decision-curve analysis (DCA) was applied to evaluate the clinical applicability of the constructed nomogram by quantifying the net improved benefits at various threshold probabilities. All statistical analyses and plots were performed using SPSS 23.0 (Chicago, IL, USA) and R version 3.6.2 (http://www.R-projectct.org/). *P* values < 0.05 were considered significant. All methods were performed in accordance with the relevant guidelines and regulations.

## Results

### Incidence and survival analyses

**Fig. 2:**
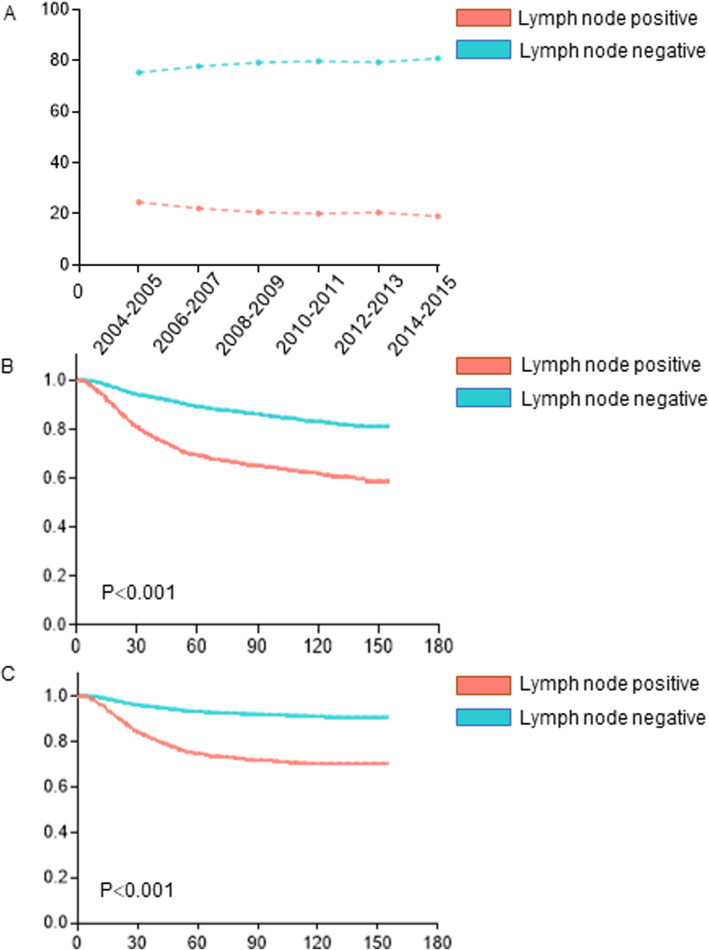
Incidence and survival analyses of lymph node metastasis. **a** Incidence of lymph node metastasis; Kaplan-Meier survival curves of OS (**b**) and CSS (**c**) showing the association between lymph node metastasis and prognosis

From 2004 to 2015, 1638 patients with cervical cancer exhibited pelvic lymph node metastasis (21.17%). First, the incidence of lymph node metastasis in patients with cervical cancer decreased slightly from 2004 to 2015, as shown in Fig. 2a. According to the log-rank test of either OS or CSS, pelvic lymph node metastasis was a high-risk factor for the prognosis of patients with cervical cancer (both *p* < 0.001), and Kaplan-Meier survival curves also confirmed the effect of lymph node metastasis on the prognosis, as shown in Fig. 2b-c.

### Patients characteristics

The training cohort included, a total of 1683 patients diagnosed with cervical cancer between 2004 and 2015. For the validation cohort, 148 patients met the criteria in the First Affiliated Hospital of Anhui Medical University between 2009 and 2019, 125 of whom were in active follow-up, and 15.54% were lost to follow-up. The majority of patients in the training cohort were middle-aged women (57.80%), and patients in the validation cohort were middle-aged women, similar to the training cohort. Regarding tumour characteristics, squamous cell carcinoma, confined to the cervix uteri / uterus and with tumour size ≥4 was the main diagnosis in both cohorts. However, the distributions of poor differentiated tumours distributions were 52.70, and 22.40% respectively in training and validation cohorts, respectively. Details are provided in Table 1. The OS rate of the training and validation cohorts were 30.30, 32.80%, respectively. The CSS rates of the training and validation cohorts were 23.10, 25.60%, respectively.

**Table 1:** Patient characteristics in the training cohort and validation cohort

	Training cohort(*N* = 1683)		Validation cohort(*N* = 125)	
	N	Percent	N	Percent
Age				
X < 30	132	7.80	3	2.40
30 ≤ X < 50	972	57.80	82	65.60
50 ≤ X < 70	494	29.40	40	32.00
X ≥ 70	85	5.10	0	0.00
Race				
White	1334	79.30	0	0.00
Black	134	8.0	0	0.00
Others	215	12.80	125	100.00
Marital status				
Married	834	49.60	112	89.60
Single	518	30.80	0	0.00
Divorced / Separated	238	14.10	7	5.60
Widowed	93	5.50	6	4.80
Histology				
Squamous cell carcinoma	1211	72.00	99	79.20
Adenocarcinoma	337	20.00	25	20.00
Adenosquamous	135	8.00	1	0.80
Differentiation				
Well differentiation	79	4.70	4	3.20
Moderate differentiation	717	42.60	93	74.40
Poor differentiation	887	52.70	28	22.40
Extension range				
Confined to the cervix uteri / uterus	1036	61.60	91	72.80
Extension beyond uterus	647	38.40	34	27.20
Tumour size (cm)				
X < 2	214	12.70	6	4.80
2 ≤ X < 4	623	37.00	47	37.60
X ≥ 4	846	50.30	72	57.60
Radiotherapy				
Yes	1433	85.10	92	73.60
No	250	14.90	33	26.40
Chemotherapy				
Yes	1337	79.40	116	92.80
No	346	20.60	9	7.20
Surgery				
Preserve uterus	341	20.30	0	0.00
Hysterectomy	1342	79.70	125	100.00
Survival state				
Survive	1173	69.70	84	67.20
Dead of other cause	121	7.20	9	7.20
Dead of cervical cancer	389	23.10	32	25.60

### Prognostic factors for OS and CSS

**Fig. 3:**
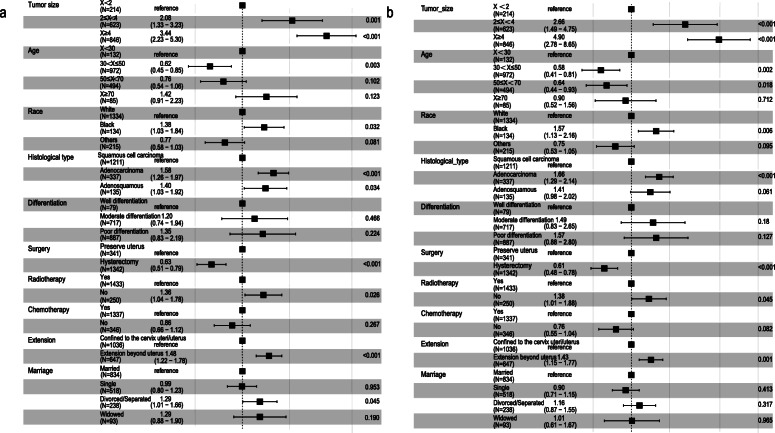
Forest plots of the multivariate analysis of OS (**a**) and CSS (**b**) in patients with stage IIIC1 cervical cancer

Univariate and multivariate Cox proportional hazard regression analyses were used to calculate the prognostic factors for OS and CSS. The results of the univariate Cox proportional hazard regression analysis are shown in Table 2, and the results of the multivariate Cox proportional hazard regression analysis are shown in forest plots (Fig. 3a-b). Age, race, marriage status, histology, extension range, tumour size, radiotherapy and surgery were all independent prognostic factors for OS. Independent prognostic factors for CSS were the same as those for OS, except the marriage status.

**Table 2:** Univariate analysis of OS and CSS in the training cohort

Characteristics	OS		CSS	
	Hazard ratios (95% CI)	P	Hazard ratios (95% CI)	P
Age				
X < 30	Reference		Reference	
30 ≤ X < 50	0.628 (0.461–0.855)	0.003	0.597 (0.427–0.835)	0.003
50 ≤ X < 70	0.859 (0.622–1.187)	0.358	0.722 (0.506–1.031)	0.073
X ≥ 70	1.737 (1.167–2.587)	0.007	1.025 (0.623–1.686)	0.922
Race				
White	Reference		Reference	
Black	1.363 (1.025–1.810)	0.033	1.512 (1.106–2.067)	0.010
Others	0.835 (0.628–1.109)	0.212	0.773 (0.552–1.083)	0.134
Marriage				
Married	Reference		Reference	
Single	1.089 (0.887–1.337)	0.415	1.030 (0.818–1.297)	0.802
Divorced / Separated	1.369 (1.071–1.751)	0.012	1.193 (0.897–1.588)	0.225
Widowed	1.820 (1.301–2.546)	< 0.001	1.161 (0.740–1.823)	0.516
Histology				
Squamous cell carcinoma	Reference		Reference	
Adenocarcinoma	1.285 (1.043–1.581)	0.018	1.345 (1.063–1.701)	0.014
Adenosquamous	1.284 (0.944–1.746)	0.112	1.298 (0.912–1.848)	0.147
Differentiation				
Well differentiation	Reference		Reference	
Moderate differentiation	1.133 (0.707–1.815)	0.603	1.326 (0.753–2.335)	0.328
Poor differentiation	1.406 (0.884–2.236)	0.150	1.524 (0.871–2.667)	0.140
Extension range				
Confined to the cervix uteri / uterus	Reference		Reference	
Extension beyond uterus	2.044 (1.718–2.432)	< 0.001	2.013 (1.650–2.456)	< 0.001
Tumour size (cm)				
X < 2	Reference		Reference	
2 ≤ X < 4	2.323 (1.498–3.602)	< 0.001	2.933 (1.650–5.214)	< 0.001
X ≥ 4	4.574 (2.998–6.980)	< 0.001	6.407 (3.672–11.180)	< 0.001
Radiotherapy				
Yes	Reference		Reference	
No	1.314 (1.047–1.651)	0.019	1.232 (0.944–1.608)	0.125
Chemotherapy				
Yes	Reference		Reference	
No	0.906 (0.729–1.126)	0.374	0.778 (0.599–1.011)	0.060
Surgery				
Preserve uterus	Reference		Reference	
Hysterectomy	0.537 (0.443–0.652)	< 0.001	0.504 (0.405–0.627)	< 0.001

### Nomograms

**Fig. 4:**
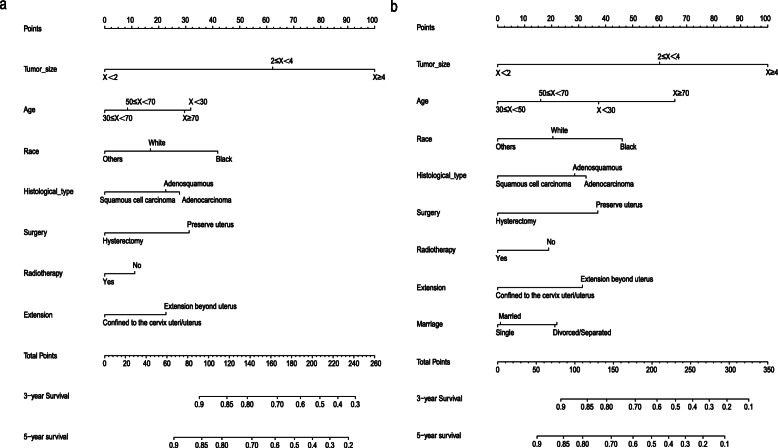
Nomogram predicting the 3- and 5-year OS (**a**) and CSS (**b**) in patients with stage IIIC1 cervical cancer

**Fig. 5:**
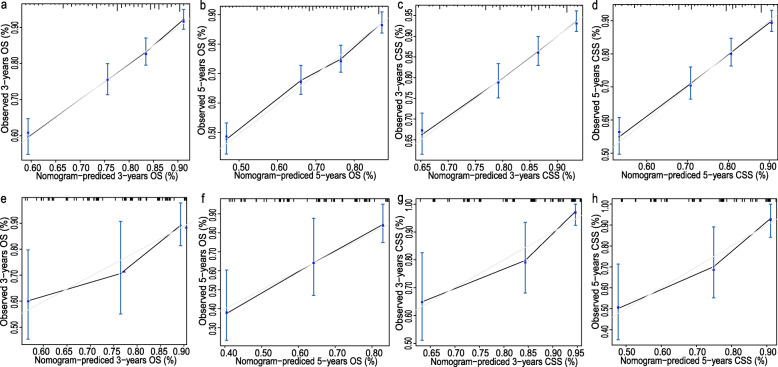
Calibration plots for (**a**) the 3-year OS nomogram in the training cohort; **b** the 5-year OS nomogram in the training cohort; **c** the 3-year CSS nomogram in the training cohort; **d** the 5-year CSS nomogram in the training cohort; **e** the 3-year OS nomogram in the validation cohort; **f** the 5-year OS nomogram in the validation cohort; **g** the 3-year CSS nomogram in the validation cohort; **h** the 5-year CSS nomogram in the validation cohort

**Fig. 6:**
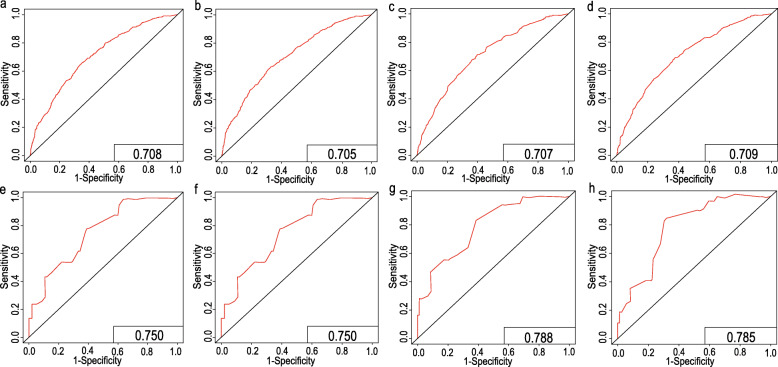
ROC curves for (**a**) the 3-year OS nomogram in the training cohort; **b** the 5-year OS nomogram in the training cohort; **c** the 3-year CSS nomogram in the training cohort; **d** the 5-year CSS nomogram in the training cohort; **e** the 3-year OS nomogram in the validation cohort; **f** the 5-year OS nomogram in the validation cohort; **g** the 3-year CSS nomogram in the validation cohort; **h** the 5-year CSS nomogram in the validation cohort

**Fig. 7:**
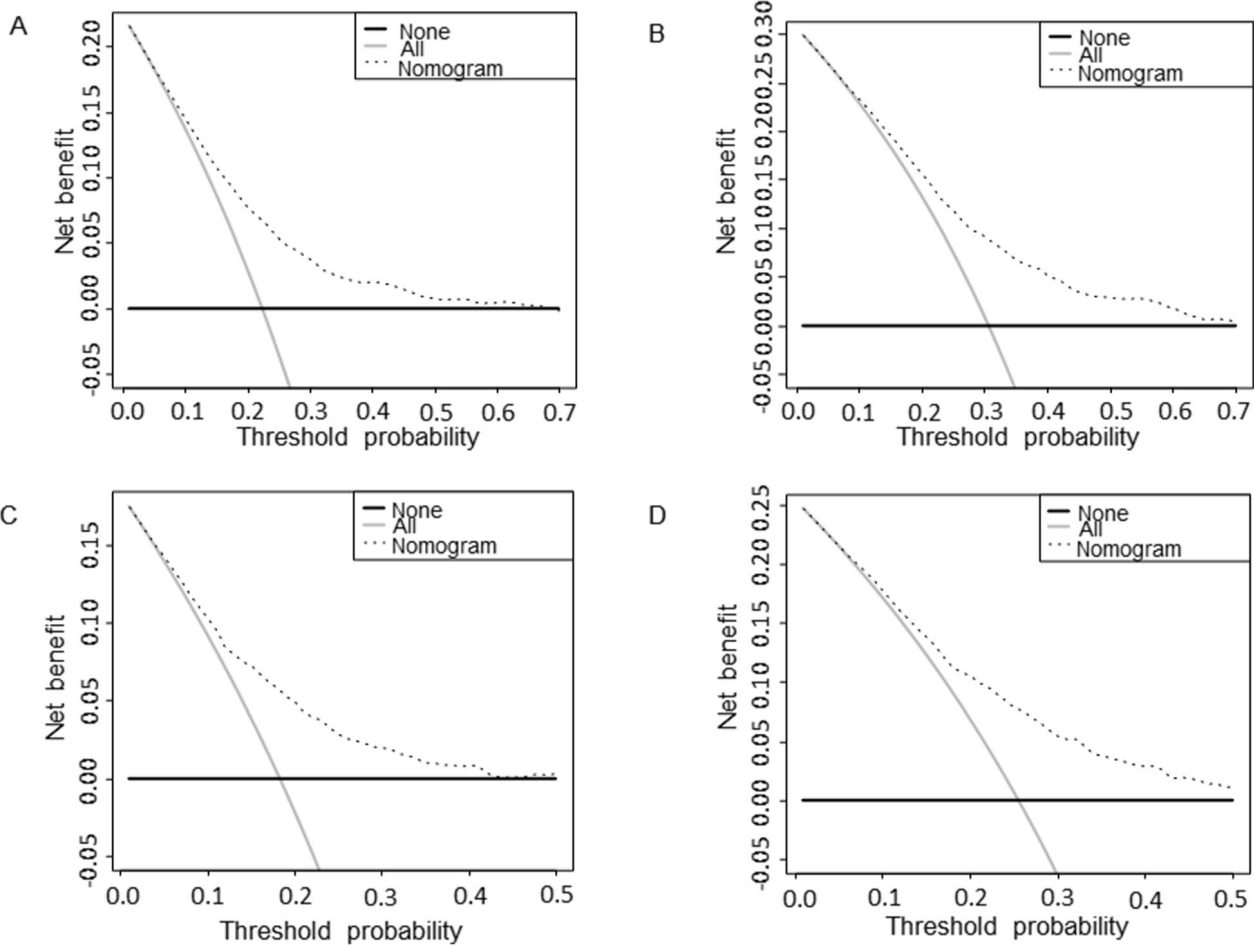
Decision curves for (**a**) predicting 3-year OS nomogram; **b** predicting 5-year OS nomogram; **c** predicting 3-year CSS nomogram; **d** predicting 5-year CSS nomogram

Based on the prognostic factors for OS and CSS derived from the Cox proportional hazard regression analyses, OS and CSS nomograms were established and are shown in Fig. 4. The C-indexes for the nomograms of OS and CSS were 0.687 and 0.692, respectively, using random sampling of SEER data sets and 0.701 and 0.735, respectively, when random sampling of external data sets. Calibration plots for the nomograms showed that the predicted 3- and 5-year OS and CSS probabilities for the training and validation sets were almost identical to the actual observations, as displayed in Fig. 5. As shown in the ROC curves for the nomogram prediction models presented in Fig. 6, the 3- and 5-year AUCs for the nomogram of OS were 0.708 and 0.705, respectively, for SEER data sets and 0.750 and 0.750, respectively, for external data sets. In addition, AUCs were 0.707 and 0.709 for the nomogram of CSS when validated using SEER data sets, and 0.788 and 0.785 when validated using external data sets. All the AUCs indicated a good discrimination ability of the model. The DCA also showed the value of the two models. The net benefit of our prognostic models was larger than that in the other two scenarios (all screening or nonscreening) in a wide range of threshold probabilities as displayed in Fig. 7.

## Discussion

Researchers have discovered that pelvic lymph node metastasis is a high-risk factor for patients with cervical cancer, which has been confirmed in many studies [13, 14]. Until 2018, FIGO agreed that lymph node metastasis had the greatest effect on the prognosis of patients diagnosed with cervical cancer, except for spreading to adjacent pelvic organs or distant organs. However, a study by Xiaoliang Liu found that the survival rate is heterogeneous in patients with stage IIIC1 cervical cancer**,** and tumour size, extension range, and other factors exert significant effects on the prognosis of stage IIIC1 cervical cancer [15]. Therefore, we included 10 variables from the SEER database to analyse the factors that affect the prognosis of patients with stage IIIC1 cervical cancer. Furthermore, previous studies documented that these 10 variables were significantly associated with the prognosis of cervical cancer. For this reason, univariate and multivariate Cox proportional hazard regression analyses were performed for all these 10 variables [15–17].

Then, we established OS and CSS nomograms based on the results of the multivariate Cox proportional hazard regression analysis. The factors in the OS nomogram included age, race, marriage status, tumour size, histology, extension range, surgery, and radiotherapy. For the CSS nomogram, only marriage status was excluded. Previous studies of the effect of marital status on cancer have shown that married patients have advantages in terms of the early diagnosis of cancer, which included cervical cancer [18]. In addition, married patients are able to receive more comprehensive adjuvant treatment, leading to a better prognosis of cervical cancer [19]. However, we found that marriage status was not an independent prognostic factor for CSS in patients with stage IIIC1 cervical cancer. Notably, unmarried patients also had diabetes, hypertension, cardiovascular disease and other comorbidities [20]. These additional diseases increase the risk of patients died from other causes and may be the main reason why marriage status was not an independent prognostic factor for CSS in patients with stage IIIC1 cervical cancer.

Our nomograms are highly innovative and practical. First, although nomograms for cervical cancer have been widely used [17, 21], a nomogram for stage IIIC1 cervical cancer is not available. Second, in contrast to the FIGO stage, patient demographics (age, race and marriage status), tumour characteristics (tumour size, histology, and extension range) and treatment (surgery and radiotherapy) which were independent prognostic factors for OS or CSS were included in our nomograms. And these variables are easily obtained in the clinic. Therefore, our nomograms could reduce the bias caused by patient demographics and different treatments when predicting the prognosis of stage IIIC1 cervical cancer compare to the FIGO stage. Third, our nomograms were verified using external data sets. This process can test the predictive ability of the nomogram in different groups of people and judge its applicability to various groups of people [22].

The C-indexes of the nomograms of random sampling of SEER and external data sets were all range from 0.65 to 0.75, which were acceptable and indicating that our nomograms have favourable discrimination ability [23, 24]. In addition, our calibration plots fit well with the 45-degree line, which means our nomograms have a fine calibration [25]. Therefore, our nomograms have good calibration in predicting 3- and 5-year OS and CSS. And DCA was performed to evaluate the clinical applicability of the constructed nomograms when quantifying the net improvement benefits under different threshold probabilities [26]. After validation, DCA confirmed that our nomograms have better clinical benefits and utility in predicting the survival of patients with stage IIIC1 cervical cancer.

Notably, tumour size had the largest contribution to the OS and CSS nomograms.. In cervical cancer, the effect of tumor size on prognosis in stage IB and stage II has been confirmed and shown in the FIGO staging system [27, 28]. According to the multivariate Cox proportional hazard regression analysis, as the tumour size increases, the prognosis of patients with stage IIIC1 disease becomes significantly worse. Meanwhile, imaging data can provide evidence of the FIGO stage [7]. Studies on the application of imaging to assess the tumour size of cervical cancer before surgery show that the diagnostic power of imaging is obviously stronger than a clinical assessment [29], especially MRI, depending on its superior contrast resolution, which can visualize the tumour volume and size [30]. We conclude that compared to other pathological characteristics, the effect of tumour size on the prognosis of cervical cancer is consistent across most stages. Further research revealed the value of tumour size as a prognostic indicator of stage IIIC1 cervical cancer. Therefore, we suggest that stage IIIC1 cervical cancer should be further divided into three substages and treated with different strategies according to tumour size.

Although the nomograms were verified using an external data set, our study still has some limitations. First, as a retrospective study, this research filtered data from data sets and excluded patients with missing data for the collected variables, leading to selection bias. Second, some key indicators are lacking, especially the dosage of radiotherapy and details of chemotherapy regimens. For example, only “Yes” and “No” were shown in the SEER database for chemotherapy, leading to a weaken effect of chemotherapy on survival. Third, the insufficient sample size of the external data set and some missing data caused inadequate verification.

## Conclusions

In conclusion, age, race, marriage status, histology, extension range, tumour size, radiotherapy and surgery were all independent prognostic factors for OS. The same factors predicted CSS, with the exception of the marriage status. In addition, OS and CSS nomograms were established in our study based on the results of a multivariate Cox proportional hazard regression analysis, and both had good predictive and clinical application value after validation. Notably, tumour size had the largest contribution to the OS and CSS nomograms.

## Data Availability

One of the data of this study are available from the Surveillance, Epidemiology, and End Results (SEER) database (https://seer.cancer.gov/). But the availability of these data was restrictive, which were used under license for the current study (ID: 10086-Nov2019), and so are not publicly available. Based on reasonable request and with permission of SEER database, data are available from the corresponding authors. The external data were obtained from the First Affiliated Hospital of Anhui Medical University.

## Abbreviations

FIGO: International Federation of Obstetrics and Gynecology
SEER: The Surveil-lance, Epidemiology, and End Results
OS: Overall Survival
CSS: Cancer Special Survival
HR: Hazard Ratio
CIs: Confidence Intervals
ROC: Receiver Operating Characteristic
DCA: Decision-Curve Analysis
